# Complete genome sequences of four *Ochrobactrum pituitosum* strains and three *Pseudochrobactrum saccharolyticum* strains isolated from *Caenorhabditis elegans* gut microbiomes

**DOI:** 10.1128/mra.00833-25

**Published:** 2026-04-21

**Authors:** Sandhya Lakshmi Narayanan, Carsten Fortmann-Grote, Paul B. Rainey

**Affiliations:** 1 Department of Microbial Population Biology, Max Planck Institute for Evolutionary Biology 28319, Plön, Germany; 2 Laboratory of Biophysics and Evolution, CBI, ESPCI Paris, Université PSL, CNRS 316338, Paris, France; Nanchang University, Nanchang, Jiangxi, China

**Keywords:** *Ochrobactrum*, *Pseudochrobactrum*, assembly, annotation, environmental sample, *C. elegans*, compost

## Abstract

We report complete genomes of four strains of *Ochrobactrum pituitosum* and three strains of *Pseudochrobactrum saccharolyticum* isolated from gut microbiomes of *Caenorhabditis elegans* colonized with microbial communities extracted from garden compost. Genomes were assembled from Oxford Nanopore long read data and polished with Illumina short read data.

## ANNOUNCEMENT

We announce closed genome assemblies and annotations of four *Ochrobactrum pituitosum* strains (NCBI: txid571256) and three *Pseudochrobactrum saccharolyticum* strains (NCBI: txid354352). Both taxa are part of the Family Brucellaceae (NCBI: txid118882).

Strains were isolated from the gut microbiome of *Caenorhabditis elegans* strain N2 (Caenorhabditis Genomic Center) (1). Three microbial communities were extracted from garden compost, frozen at −80°C, re-thawed, and acclimatized on plates (M9 buffer, 1.5% agar) containing 4 cm^2^ cellulose paper at 20°C. After 2 weeks, community-degraded paper was suspended in buffer, and 100 µL of the slurry was transferred to an identical piece of paper on fresh agar. Approximately 100 nematodes were added to the paper, with nematodes feeding leading to the colonization of the gut by bacteria.

After three 2-week transfers, nematodes were collected from the paper, surface-sterilized, and pulverized for 5 min using a tissue lyser (TissueLyser II, Qiagen) for microbiome extraction. Extracted samples were serially diluted and plated on TSA agar plates and incubated at 30°C for 24–48 h. Single colonies were purified by re-streaking colonies on TSA plates and stored in 70% glycerol saline aliquots at −80°C. Samples were thawed and re-grown (overnight) in shaking 6 mL LB broth at 28°C. Genomic DNA was extracted following the method described in a previous study (2) and purified using the Genomic DNA Clean & Concentrate kit–10 (Zymo Research). DNA was quantified using the Invitrogen Qubit dsDNA Broad Range (BR) assay kit.

Long-read sequencing (Oxford Nanopore Technology) was performed by Eurofins (Germany). Short-read sequencing was performed on a NextSeq500 platform (MPI for Evolutionary Biology) at 150 bp read length, paired-end sequencing, and 60-fold coverage. In-house library preparation used a Tn5 transposase enzyme library (3). Short-read raw data were quality-controlled using MultiQC v1.11 (4). Here, and in the following, default parameters were applied unless otherwise noted.

Raw nanopore sequencing (5) was filtered for low quality and short reads using Filtlong v0.2.1 (6) before being assembled using Flye v2.9.3 (7); parameters were optimized for bacterial genomes (7). Contigs were polished using Medaka v1.8 (8). Assembled genomes were annotated using Bakta v.1.8.2 (9). Quality of the assembled genomes was assessed using QUAST v5.2 (10), CheckM2 v1.0.1 (11), and Mash v2.3 (12). As a final QC step, sequence-cleaned reads were mapped onto the assembly using minimap2 v2.24 (13). Finally, long-read assemblies were polished with our Illumina short reads using nextpolish v1.4.1 (14) and postprocessed with samtools v1.2.1 (15).

Assembled genomes were circularized with DNApler (16) (default parameters) such that the start site coincides with the dnaA gene start codon. Average genome coverage was 84.0× with a GC content of 57.8%.

Genera were identified by 16S metabarcode sequencing (using primers 341F (CCTACGGGNGGCWGCAG) and 805R (GACTACHVGGGTATCTAATCC) to amplify the hypervariable V3-V4 region) and analyzed using phylos v1.52.0 (17). Isolates were inferred from a gyrA neighbor-joining phylogeny (Tamura-Nei distance) using ClustalΩ v1.2.4 (1, 18).

**Fig 1:**
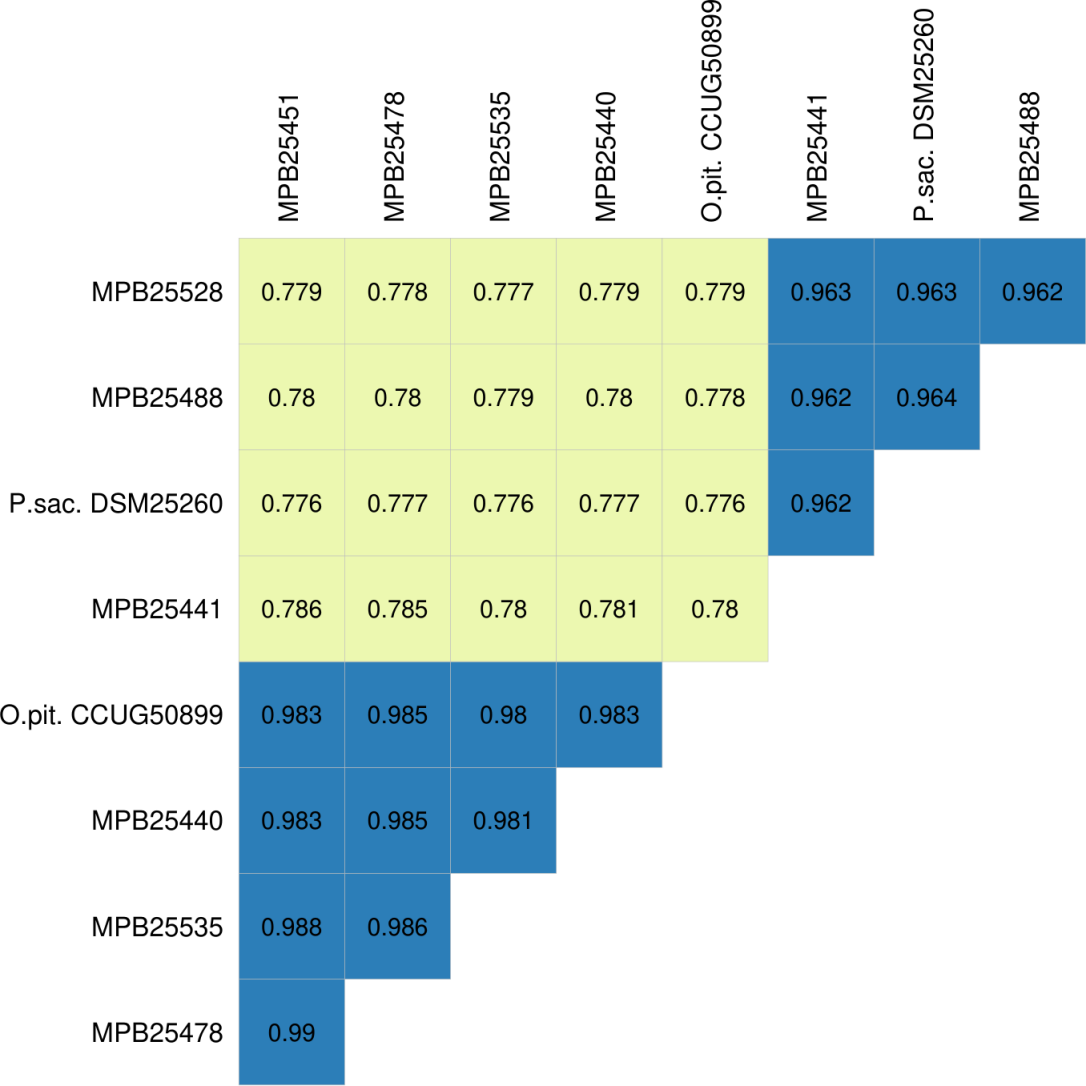
Clustered heatmap of ANI similarity values for seven announced strains and type strains *P. saccharolyticum* DSM25260 and *O. pituitosum* CCUG50899.

Strains taxonomy was determined by ANI analysis (19, 20), including type strains *Ochrobactrum pituitosum* CCUG:50899 and *Pseudochrobactrum saccharolyticum* DSM:25620, using FastANI v0.1.3 (21) (Fig. 1). All samples,raw reads, assemblies, and annotations are deposited on the ENA, see Table 1.

**TABLE 1:** Summary table

Sample	Property	Value
SAMEA118333800	Organism	*O. pituitosum*
	Taxon ID	71256
	Isolate/strain	MPB25535
	Short read exp	ERX14406277
	Short read run	ERR15002230
	Short read count	1,727,681
	Short read GC%	53.3
	Short read mean length (Rl, R2)	150,150
	Long read exp	ERX14512245
	Long read run	ERR15107547
	Long read count	87,517
	Long read mean length	6113
	Long read GC%	55.0
	Assembly	GCA_965577835
	Total size	4,963,799
	Contigs	3
	GC%	53.4
	N50	13,839,640
	ANI (*O. pituitosum* CCUG50899)	98.06
	Annotation	ERZ26904280
	Annotated CDSs	4662
SAMEA118333801	Organism	*O. pituitosum*
	Taxon ID	71256
	Isolate/Strain	MPB25451
	Short read exp	ERX14406278
	Short read run	ERR15002231
	Short read count	1,790,809
	Short read mean length (R1, R2)	150,150
	Short read GC%	53.5
	Long read exp	ERX14512247
	Long read run	ERR15107549
	Long read count	50,100
	Long read mean length	4595
	Long read GC%	51.8
	Assembly	GCA_965579405
	Total size	5,097,185
	Contigs	3
	GC%	53.5
	N50	6,950,440
	ANI (*O. pituitosum* CCUG50899)	98.27
	Annotation	ERZ26904286
	Annotated CDSs	4825
SAMEA118333802	Organism	*O. pituitosum*
	Taxon ID	71256
	Isolate/Strain	MPB25440
	Short read exp	ERX14406279
	Short read run	ERR15002232
	Short read count	1,595,120
	Short read mean length (Rl, R2)	150,150
	Short read GC%	53.5
	Long read exp	ERX14512249
	Long read run	ERR15107551
	Long read count	44,497
	Long read mean length	6424
	Long read GC%	53.5
	Assembly	GCA_965579335
	Total size	4,550,329
	Contigs	4
	GC%	53.5
	N50	7959060
	ANI (*O. pituitosum* CCUG50899)	98.40
	Annotation	ERZ26904287
	Annotated CDSs	4285
SAMEA118333803	Organism	*O. pituitosum*
	Taxon ID	71256
	Isolate/Strain	MPB25478
	Short read exp	ERX14512100
	Short read run	ERR15107402
	Short read count	1,630,005
	Short read mean length (R1, R2)	150,150
	Short read GC%	53.3
	Long read exp	ERX14512251
	Long read run	ERR15107553
	Long read count	74,814
	Long read mean length	3862
	Long read GC%	53.3
	Assembly	GCA_965579345
	Total size	4,852,914
	Contigs	4
	GC%	53.3
	N50	39,450,584
	ANI (*O. pituitosum* CCUG50899)	98.52
	Annotation	ERZ26904288
	Annotated CDSs	4552
SAMEA118333804	Organism	*P. saccharolyticum*
	Taxon ID	54352
	Isolate/Strain	MPB25528
	Short read exp	ERX14406336
	Short read run	ERR15002289
	Short read count	440,692
	Short read mean length (Rl, R2)	150,150
	Short read GC%	51.5
	Long read exp	ERX14512252
	Long read run	ERR15107554
	Long read count	48,318
	Long read mean length	5292
	Long read GC%	56.3
	Assembly	GCA_965579315
	Total size	3,733,418
	Contigs	3
	GC%	51.5
	N50	12184706
	ANI (*P. saccharolyticum* DSM25260)	96.29
	Annotation	ERZ26904290
	Annotated CDSs	3348
SAMEA118333805	Organism	*P. saccharolyticum*
	Taxon ID	54352
	Isolate/Strain	MPB25488
	Short read exp	ERX14406304
	Short read run	ERR15002257
	Short read count	419,074
	Short read mean length (Rl, R2)	150,150
	Short read GC%	51.4
	Long read exp	ERX14512253
	Long read run	ERR15107555
	Long read count	33,095
	Long read mean length	6,247
	Long read GC%	55.1
	Assembly	GCA_965579355
	Total size	3,753,639
	Contigs	2
	GC%	51.5
	N50	10,475,352
	ANI (*P. saccharolyticum* DSM25260)	96.35
	Annotation	ERZ26904289
	Annotated CDSs	3365
SAMEA118333806	Organism	*P. saccharolyticum*
	Taxon ID	54352
	Isolate/Strain	MPB25441
	Short read exp	ERX14406305
	Short read run	ERR15002258
	Short read count	478,435
	Short read mean length (R1, R2)	150,150
	Short read GC%	51.6
	Long read exp	ERX14512255
	Long read run	ERR15107557
	Long read count	149,774
	Long read mean length	5140
	Long read GC%	54.7
	Assembly	GCA_965579305
	Total size	3,839,354
	Contigs	2
	GC%	51.6
	N50	5,324,660
	ANI (*P. saccharolyticum* CCUG33852)	96.20
	Annotation	ERZ26904291
	Annotated CDSs	3473

###  

## Data Availability

Samples and data were submitted to the ENA BioProject PRJEB87311, see Table 1 for accessions. Code for gyrA and ANI analysis is deposited in https://archive.softwareheritage.org/swh:1:dir:067262a26b6b51b384fe7955736135216cbbca5d.
